# Deep Learning Based on Automated Breast Volume Scanner Images for the Diagnosis of Breast Lesions: A Multicenter Diagnostic Study

**DOI:** 10.7150/ijms.118430

**Published:** 2025-08-22

**Authors:** Hui Liu, Ying Zhang, Bin Tan, Yi-Fei Yin, Li-Xia Yan, Li-Hua Xiang, Dan-Dan Shan, Yun-Yao Zhang, Shi-Si Ding, Guang Xu, Bo-Yang Zhou, Yi-Lei Shi, Xiao-Xiang Zhu, Jing-Liang Hu, Li-Ping Sun, Hui-Xiong Xu, Yi-Feng Zhang

**Affiliations:** 1 Department of Medical Ultrasound, Center of Minimally Invasive Treatment for Tumor, Shanghai Tenth People's Hospital, School of Medicine, Tongji University, Shanghai 200072, China.; 2 Ultrasound Research and Education Institute, Clinical Research Center for Interventional Medicine, School of Medicine, Tongji University Shanghai 200072, China.; 3 MedAI Technology (Wuxi) Co. Ltd, Wuxi, China.; 4 Department of Medical Ultrasound, Affiliated Hospital of Nantong University, Nantong, 226001, China.; 5 Department of Ultrasound, Institute of Ultrasound in Medicine and Engineering, Zhongshan Hospital, Fudan University, Shanghai 200032, China.; 6 Chair of Data Science in Earth Observation, Technical University of Munich, Germany.; 7 Department of Ultrasound, Shanghai General Hospital, Shanghai Jiao Tong University School of Medicine, Shanghai 200080, China.

**Keywords:** Breast cancer, Automated breast volume scanner, Ultrasound, Deep learning.

## Abstract

**Objectives:** To develop a deep learning (DL) model for the automated detection and diagnosis of breast cancer utilizing automated breast volume scanner (ABVS) images, and to compare its diagnostic performance with that of radiologists in screening ABVS examinations.

**Methods:** In this multicenter diagnostic study, ABVS data from 1,368 patients with breast lesions were collected across three hospitals between November 2019 and April 2024. The DL model (VGG19, DenseNet161, ResNet101, and ResNet50) was developed to detect and classify lesions. One-tenth of the cases from Hospital A were randomly selected as a fixed internal test set; the remaining data were randomly divided into training and validation sets at an 8:2 ratio. External test sets were derived from Hospitals B and C. Pathological findings served as the gold standard. Clinical applicability was assessed by comparing radiologists' diagnostic performance with and without DL model assistance.

**Results:** For breast cancer detection, the DL model achieved an area under the receiver operating characteristic curve (AUC) of 0.984 (95% CI: 0.965-0.995) on the internal test set, 0.978 (95% CI: 0.951-0.994) on the external test set 1 (Hospital B), and 0.942 (95% CI: 0.902-0.978) on the external test set 2 (Hospital C). The model demonstrated significantly higher sensitivity (98.2%) and specificity (90.3%) than junior radiologists (P < 0.05), while exhibiting comparable diagnostic reliability and accuracy to senior radiologists. Interpretation time was significantly reduced for all radiologists when using the DL model (P < 0.05).

**Conclusion:** The DL model based on ABVS images significantly enhanced diagnostic performance and reduced interpretation time, particularly benefiting junior radiologists.

## Introduction

Breast cancer is one of the leading causes of cancer-related deaths among women worldwide, and its mortality rate is on the rise globally. Early diagnosis and treatment of breast cancer can contribute to reducing mortality rates 1,2. Wome n of Asian descent exhibit higher breast tissue density, which diminishes the sensitivity of conventional mammography for cancer detection 3-6. Breast ultrasound (US) has been suggested as an additional tool to mammography or as a standalone screening method to enhance the accuracy of cancer detection 7,8. However, US examination outcomes are highly operator-dependent, limited by suboptimal reproducibility and significant inter-operator variability 9.

The automated breast volume scanner (ABVS) utilizes a specialized high-frequency broadband transducer to perform automated breast scans, generating consistent, standardized, and reproducible high-resolution ultrasound images 10. Studies demonstrate that ABVS effectively overcomes the limitations inherent in handheld ultrasound (HHUS) while providing comparable diagnostic accuracy 11. This system generates three-dimensional (3D) images of breast lesions, enabling comprehensive visualization from multiple perspectives, including transverse, sagittal, and coronal planes. The incorporation of morphological features within the characteristic coronal plane has proven particularly advantageous for enhancing early detection in dense breast tissue and mitigating limitations associated with preoperative breast lesion diagnosis. When integrated with conventional mammography, ABVS has been demonstrated to improve the cancer detection rates 12,13. However, the substantial volume of images generated per ABVS scan necessitates longer interpretation times, particularly for less experienced radiologists. Furthermore, studies suggest an increased dependency on radiologist expertise for ABVS assessment 14.

In recent years, substantial advancements have been achieved in the developing convolutional neural networks (CNNs) employing deep learning (DL) algorithms for medical images analysis 15-17. DL processes raw image pixels as input and autonomously acquires complex patterns and features through class annotations, thereby constructing a comprehensive hierarchical representation of extracted information 18,19. CNNs autonomously identify salient image features and acquire classification capabilities during training, enabling the incorporation of characteristics imperceptible to human observers 20. Deep learning networks (DLNs) offer extensive utility in diagnostic imaging and predictive modeling due to their demonstrated advantages, including computational efficiency, high accuracy, and reproducible performance 21,22. The implementation of DLNs for feature extraction in Automated Breast Volume Scanner (ABVS) images has enhanced diagnostic robustness during secondary interpretation 23-25. Although prior DL studies have investigated ABVS applications, most models rely on transverse and sagittal plane imagery, with a paucity of DL methodologies leveraging ABVS coronal planes for breast cancer diagnosis.

Therefore, to establish a novel automated ultrasound diagnostic model for breast tumors, we developed a DL model for automatic lesion detection in ABVS images and differentiation between malignant and benign lesions. The model's performance was validated through internal and external testing. Furthermore, we compared its diagnostic performance with that of radiologists and evaluated its utility in enhancing radiologists' diagnostic accuracy.

## Methods

### Study design and participants

This study utilized ABVS images collected from three hospitals between November 2019 and April 2024. We obtained ABVS data for the training, validation, and internal test sets from the Breast Imaging Database at Hospital A (Shanghai Tenth People's Hospital). The external test sets were obtained from hospitals B (Zhongshan Hospital, Fudan University) and C (Affiliated Hospital of Nantong University). All patients in the study underwent HHUS and ABVS examinations, with the HHUS examinations aimed at validating the findings of the ABVS. Patients subsequently underwent biopsy or surgery within one month. Pathological findings served as the gold standard. The inclusion criteria were as follows: (1) patients aged ≥ 18 years; (2) patients whose breast lesions were evaluated with HHUS and ABVS examinations; and (3) patients whose lesions had not undergone biopsy or any treatment prior to the ABVS examination. The exclusion criteria for patients were as follows: (1) incomplete data and clinical information; (2) the features of the breast lesions could not be clearly observed due to shadows behind the nipple or poor-quality images; and (3) multiple lesions in one breast. The clinical information of the patients and the features of the breast lesions were recorded. This multicenter study was approved by the institutional review boards of the three participating centers (approval No.SHSY-IEC-4.1/19-205/0). Informed consent was waived by our Institutional Review Board due to the retrospective nature of our study. This study was registered at https://www.chictr.org.cn (No. ChiCTR2300074673).

### ABVS examinations

ABVS examinations were conducted using the ACUSON S2000 US system (Siemens Medical Solutions, Inc., Mountain View, CA, USA) with an automated 5-14-MHz linear broadband transducer (covering volumes of 15.4 × 16.8 × 6 cm), which acquired 0.5-mm thick images in the transverse plane. Image acquisition was performed by experienced technologists. Patients were positioned in the supine or lateral position with their arms above their head. The appropriate scan depth was selected based on the size of the breast to obtain a standardized ABVS image. After the examination, the axial ABVS images were sent to a dedicated workstation, where sagittal and coronal images were reconstructed automatically 26,27. Finally, the transverse, sagittal, and coronal ABVS images depicting the lesions were chosen for further image segmentation and feature extraction.

### Datasets

**Figure 1:**
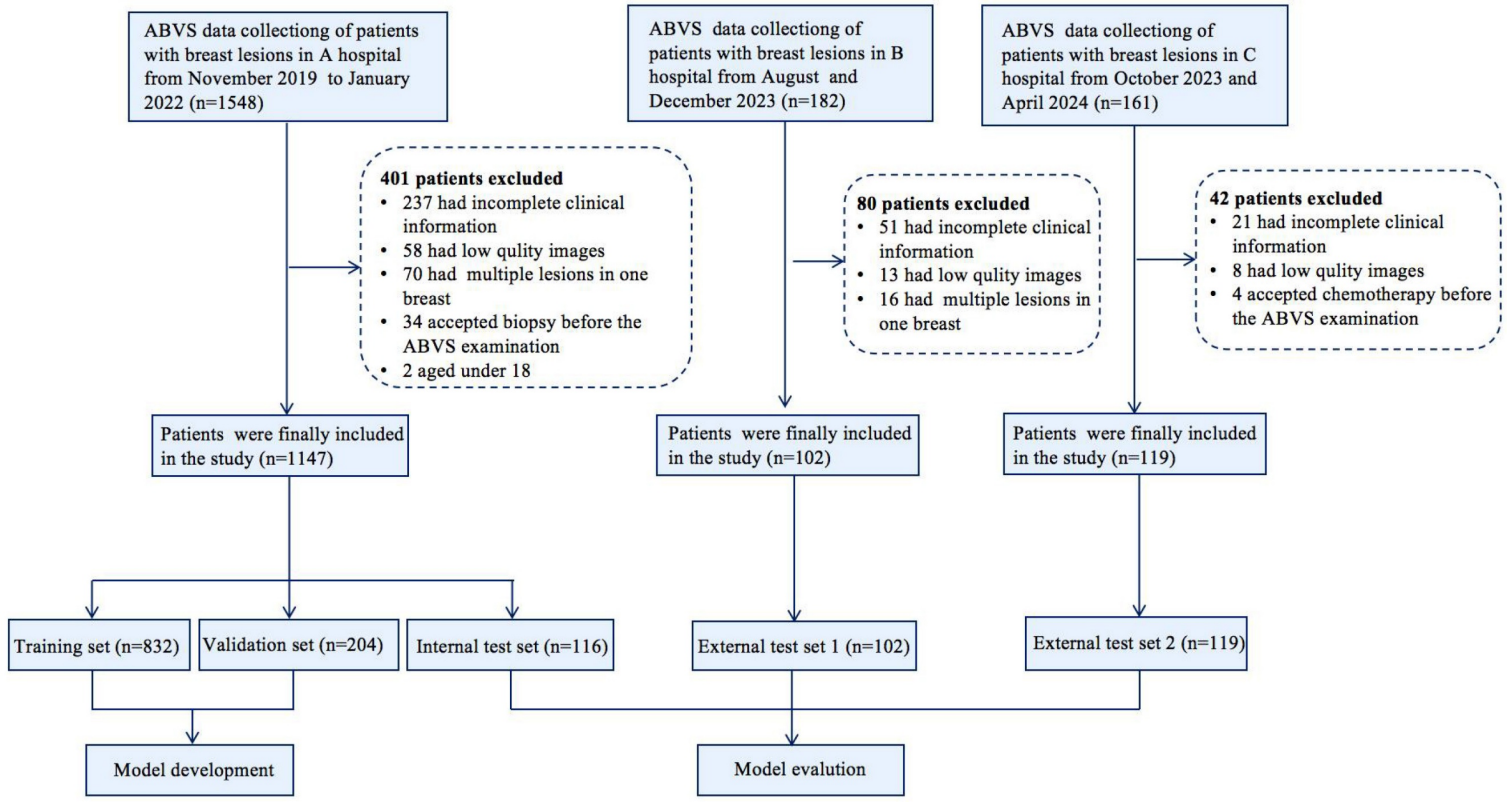
Flowchart shows the eligibility criteria and process for deep learning (DL) model development and evaluation.

Following quality control, images were acquired for the dataset from the Breast Imaging Database at three hospitals. We compiled 1,152 breast lesions with transverse, sagittal, and coronal images from 1,147 patients (aged 18-95 years) at hospital A. One-tenth of the cases were randomly selected as the fixed test set, while the remaining data were randomly divided into a training set and a validation set at a ratio of 8:2, ensuring no overlap among the three subsets. Two US radiologists with over 5 years of experience determined the boundaries and shapes of the lesions from transverse, sagittal, and coronal images and carefully marked the lesions. We obtained ABVS images for external test sets from hospitals B (102 lesions from 102 patients) and C (119 lesions from 119 patients) to assess the generalizability of the DL model. A flowchart detailing the study process is presented in Figure 1.

### Deep Learning Algorithm

**Figure 2:**
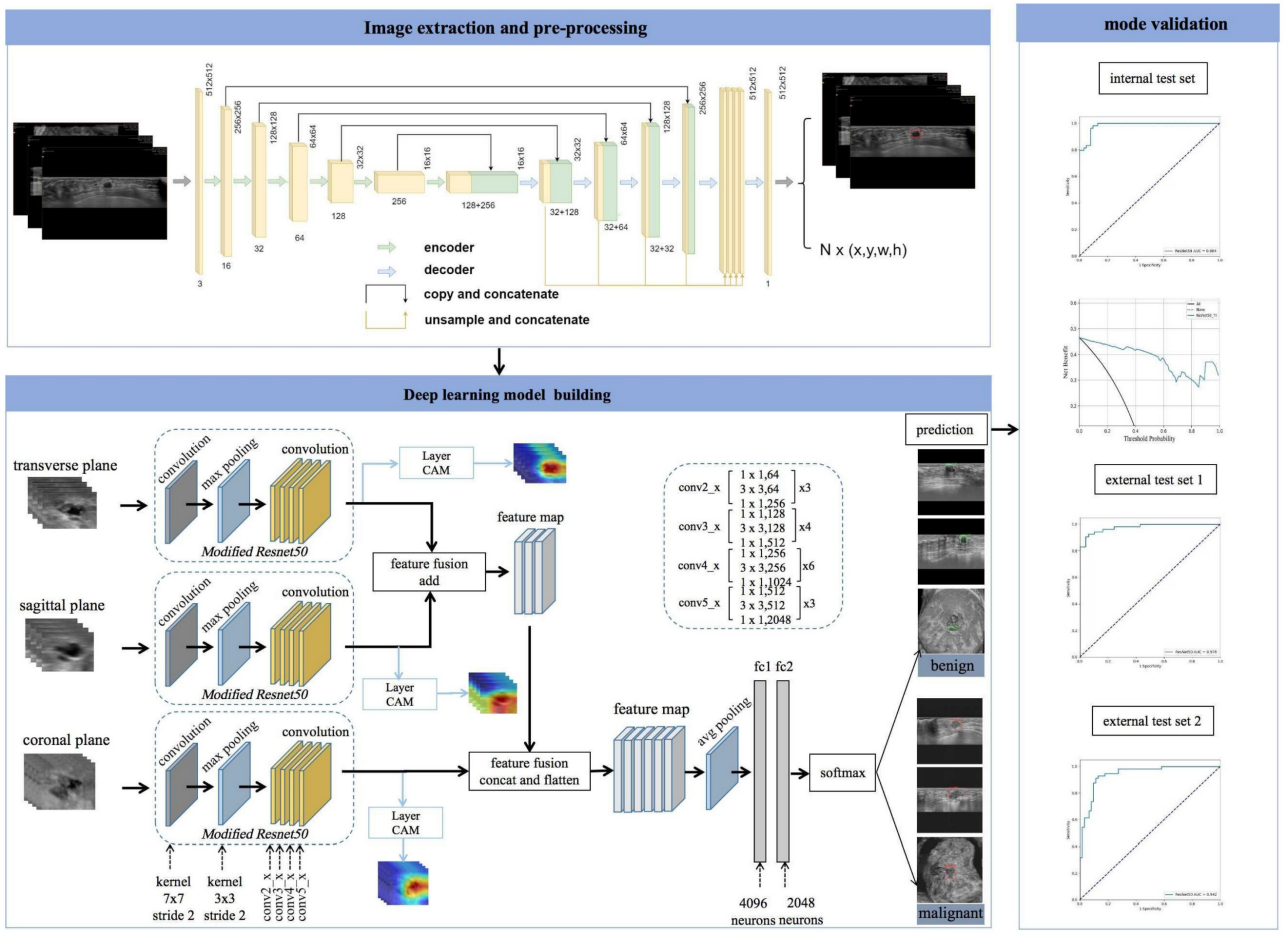
Workflow of model development and the DL neural network architecture. The model was developed utilizing multiple-planar analysis (i.e., transverse, sagittal, and coronal planes) within a DL framework. Within each processing pathway, the DL network extracts discriminative features by integrating spatial relationships via a ResNet architecture. Features aggregated from the parallel pathways were subsequently concatenated and fused by the ensuing fully connected (FC) layers. The network accepts the original image containing solely the lesion region as input and outputs the pathological binary classification alongside corresponding heatmaps visualizing salient regions.

The UNet segmentation model 28 was utilized to segment the breast lesions within image sequences across various planes. Segmentation mask images were produced, with lesions appearing white against a black background. Subsequently, the coordinates of the external rectangular frame and the regional map of the breast lesion at its coordinate position were extracted. The breast lesions in the ABVS images were precisely located and marked. The transverse, sagittal, and coronal breast lesion region maps, extracted by the UNet segmentation network, were fed into common classification networks (VGG19, DenseNet161, ResNet101, and ResNet50) 29. These networks extracted features from the three planes and employed various fusion methods (Figure 2).

The UNet segmentation network successfully maintains both the local details and the global context information of images during object segmentation. Through pooling and upsampling operations, the network adapts to structures at various scales. For ABVS images, this means that the network can detect and segment anatomical structures of different sizes and shapes. In this study, EfficientNet-b0^30^ was employed as the feature extraction network for the UNet segmentation network. The EfficientNet-b0 structure, incorporating innovative designs such as deep separable convolution, provides robust feature learning capabilities. In ABVS image segmentation tasks, it aids in extracting information about organizational structure, texture, and morphological features from input images. The procedure for breast lesion segmentation by UNet (EfficientNet-b0) on ABVS images of the breast is as follows.

The convolution operation of the UNet segmentation network in the encoder path: For each layer 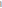, the convolution operation involves the following formula:



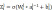



where 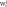
is the weight of the convolutional kernel, 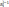
is the activation output of the previous layer, 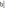
is the bias term, and 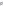
is an activation function.

The upsampling and convolution operations of the UNet segmentation network in the decoder path involve formulas such as:



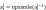





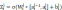



Upsampling is an operation, such as bilinear interpolation, that 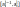
indicates the result of the previous layer's upsampling is connected with the encoder output of the corresponding layer.

The implementation of UNet (EfficientNet-b0) was based on the Segmentation Models Pytorch library [https://github.com/qubvel/segmentation_models.pytorch], which provides a modular framework for constructing architecture encoder-decoder segmentation models. The Unet segmentation network delineates and localizes breast lesions through the following procedure: the segmentation mask produced by Unet model is thresholdized to generate a binary image, wherein breast lesions are designated as foreground (white) and the background is rendered black. Connected component analysis is applied to the binary image to identify discrete regions, each representing an individual lesion object. A bounding box is subsequently computed for each connected region. This process yields both a localization map highlighting breast lesions and their corresponding spatial coordinates. These lesion coordinates enable the extraction of individual lesion regions, thereby providing input data for a subsequent classification model tasked with distinguishing benign from malignant breast lesions.

During the design of the algorithm structure, the methodology for data fusion across multiple planes was incorporated. A neural network was devised to process data from the transverse, sagittal, and coronal planes, integrating their respective feature representations to train a multimodal feature fusion network. ResNet50 was selected as the foundational architecture and subsequently refined. For each individual plane, the network was initialized using pretrained weights from ResNet50 on the ImageNet dataset. The output layer of ResNet50 was modified to comprise two nodes, corresponding to benign and malignant classifications of breast nodules. The optimizer employed Stochastic Gradient Descent (SGD) with momentum, a learning rate of 0.001, and a batch size of 64. Due to distinct variations in imaging techniques and semantic features among the transverse, sagittal, and coronal planes, feature fusion was implemented via addition for the transverse and sagittal planes, followed by concatenation with the coronal features. Equations 1 and 2 define the addition and concatenation operations, respectively.


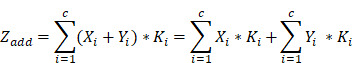

(1)


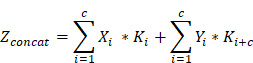

(2)

In Equation (1), X denotes the feature map of the transverse plane, Y represents the feature map of the sagittal plane, and K signifies the convolution operation to be executed subsequent to feature fusion. In Equation (2), X corresponds to the fusion feature derived from the transverse and sagittal planes, Y indicates the feature map of the coronal plane, and K represents the convolution operation post feature fusion. The parameter c denotes the number of channels in the feature map for both equations.

The concatenate feature fusion merges feature maps along the channel dimension, thereby increasing the number of features while maintaining the information content per feature. The hybrid fusion strategy combining addition and concatenation operators is employed to integrate breast features. This approach effectively addresses the limitations of low semantic richness in single-plane features and insufficient high-level breast feature representation, consequently enhancing multimodal feature fusion classification performance 31,32. Critically, concatenation reduces feature map channel dimensionality. Consequently, a fully connected layer is incorporated into the original ResNet50 architecture, reducing the dimensionality of the fused feature maps to half the original size. During model testing, a probability averaging method is applied to the prediction outcomes. The predicted benign or malignant probabilities for each image frame are summed and subsequently divided by the total number of lesion images to compute the mean probability. A lesion is classified as malignant if its malignancy probability exceeds the threshold value of 0.5; otherwise, it is classified as benign.

### Image analysis

Junior Radiologists 1 and 2, each possessing 2 years of experience in breast US diagnosis and 1 year in ABVS diagnosis; Middle Radiologists 3 and 4, each possessing 5 years of experience in breast US diagnosis and 3 years in ABVS diagnosis; and Senior Radiologists 5 and 6, each possessing 9 years of experience in breast US diagnosis and 5 years in ABVS diagnosis, participated in the study. Radiologists 1 - 6 did not perform manual lesion demarcation; they were provided solely with the primary ABVS images and corresponding case numbers, operating independently and without consultation to ensure diagnostic objectivity. Each radiologist independently reviewed the identical set of lesions and utilized the fifth edition of the Breast Imaging-Reporting and Data System (BI-RADS) lexicon 33,34 to assign a BI-RADS category based on the transverse, sagittal, and coronal image features.

### Comparative analysis with radiologists' interpretations

Employing the 5th edition of the BI-RADS classification guidelines, BI-RADS 4A denotes lesions with a low suspicion of malignancy (≤ 10% probability). This category served as the diagnostic threshold to evaluate the performance of six radiologists. Subsequently, after a one-month interval, the radiologists re-evaluated the cases incorporating DL-derived malignancy assessments (benign or malignant) to establish final diagnoses. The diagnostic performance metrics—both with and without DL assistance—alongside interpretation times, were systematically compared.

### Statistical analysis

Statistical analyses were conducted using IBM SPSS Statistics (version 27). Continuous variables were presented as the means ± standard deviations (SDs), while categorical variables were expressed as frequencies and proportions. Categorical variables were compared using the chi-square test or Fisher's exact test, and continuous variables were analyzed with t-tests. To evaluate diagnostic performance in the test set, accuracy, sensitivity, specificity, positive predictive value (PPV), negative predictive value (NPV), and area under the receiver operating characteristic curve (AUC) were calculated for both the DL model and radiologists of varying experience levels. Comparisons of sensitivity and specificity between the DL method and the six radiologists, as well as among the six individual radiologists without versus with DL assistance, were performed using McNemar's test. Reading times across radiologist groups were compared via paired t-tests. Interobserver agreement was assessed based on categorization concordance between the DL model and radiologists, with interreader correlations quantified using Fleiss' kappa coefficient (κ). The F1 score, defined as the harmonic mean of sensitivity and PPV, served as a composite performance metric; higher values indicated superior diagnostic efficacy. All evaluation metrics were reported with 95% confidence intervals (CIs). Statistical significance was defined as P < 0.05.

## Results

### Patient inclusion and grouping

Between November 2019 and January 2022, transverse, sagittal, and coronal images from 1,152 lesions of 1,147 patients were obtained from the ABVS Imaging Database at A Hospital for training (832 lesions), validation (204 lesions), and internal testing (116 lesions). Five patients presented with bilateral breast tumors, resulting in a total of ten lesions. Of the 1,152 breast lesions 535 (46.4%) were malignant, and 617 (53.6%) were benign. The maximum diameter ranged from 4-130 mm. Between August 2023 and December 2023, 102 breast lesions from 102 patients were obtained from Hospital B. Between October 2023 and April 2024, 119 breast lesions from 119 patients were obtained from Hospital C. These lesions all include transverse, sagittal, and coronal images, forming an independent external test set to fairly evaluate the DL method. The detailed patient demographics, breast lesion characteristics, and clinicopathological information for each group are summarized in Table 1.

### Performance of DL models

**Figure 3:**
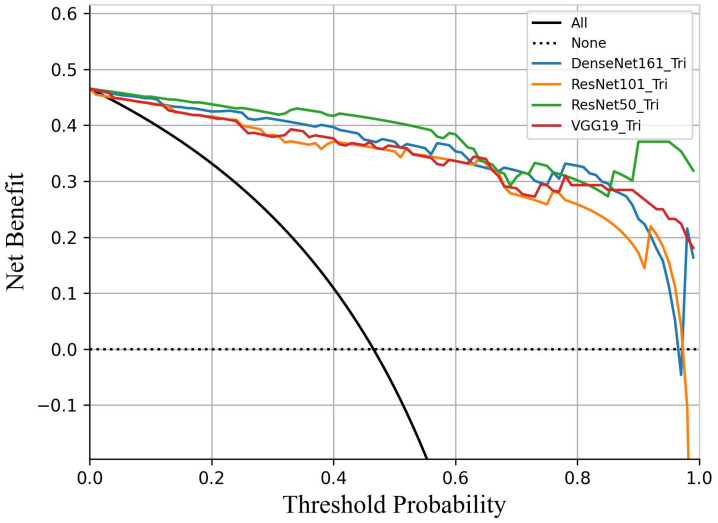
Decision curve analysis comparing the performance of four DL models in predicting breast cancer. All models demonstrated clinical utility within the threshold probability range of 45% to 95%. ResNet50 demonstrated a significantly higher net benefit than the other three models.

To simulate the clinical workflow of radiologists, who consider multiplane US images when making assessments, we merged the malignancy risk probabilities from various planes to generate an overall probability for lesion-level US imaging evaluation. Within the internal test set, we assessed the performance of the model by utilizing different plane US images, and measuring the AUC of the receiver operating characteristic curve (ROC) and F1 score (Figure 3). When the transverse, sagittal, and coronal planes were combined, ResNet50 achieved the best sensitivity (98.2%), and VGG19 performed best in terms of specificity (91.9%). Compared with the other models (VGG19, DenseNet161, ResNet101), the ResNet50 model had higher sensitivity (98.2%), NPV (98.3%), and accuracy (94.0%) (Table 2). According to the transverse and sagittal planes, the ResNet50 model achieved an AUC of 0.963. With the additional coronal planes, the model attained a significantly better AUC of 0.984. The three planes accomplished superior performance to the two planes. The evaluation of the AUC on lesion-level US images demonstrated superior performance on both two-plane and three-plane assessments compared with single-plane US images. For external test set 1, the DL model also had better sensitivity (96.2%), accuracy (90.2%), and an F1 score of 0.911. For external test set 2, the DL model also had better sensitivity (93.0%) and an F1 score of 0.876. Table 3 summarizes the statistical comparisons among various planes.

### Visualization and auxiliary diagnosis functions of the DL model

**Figure 4:**
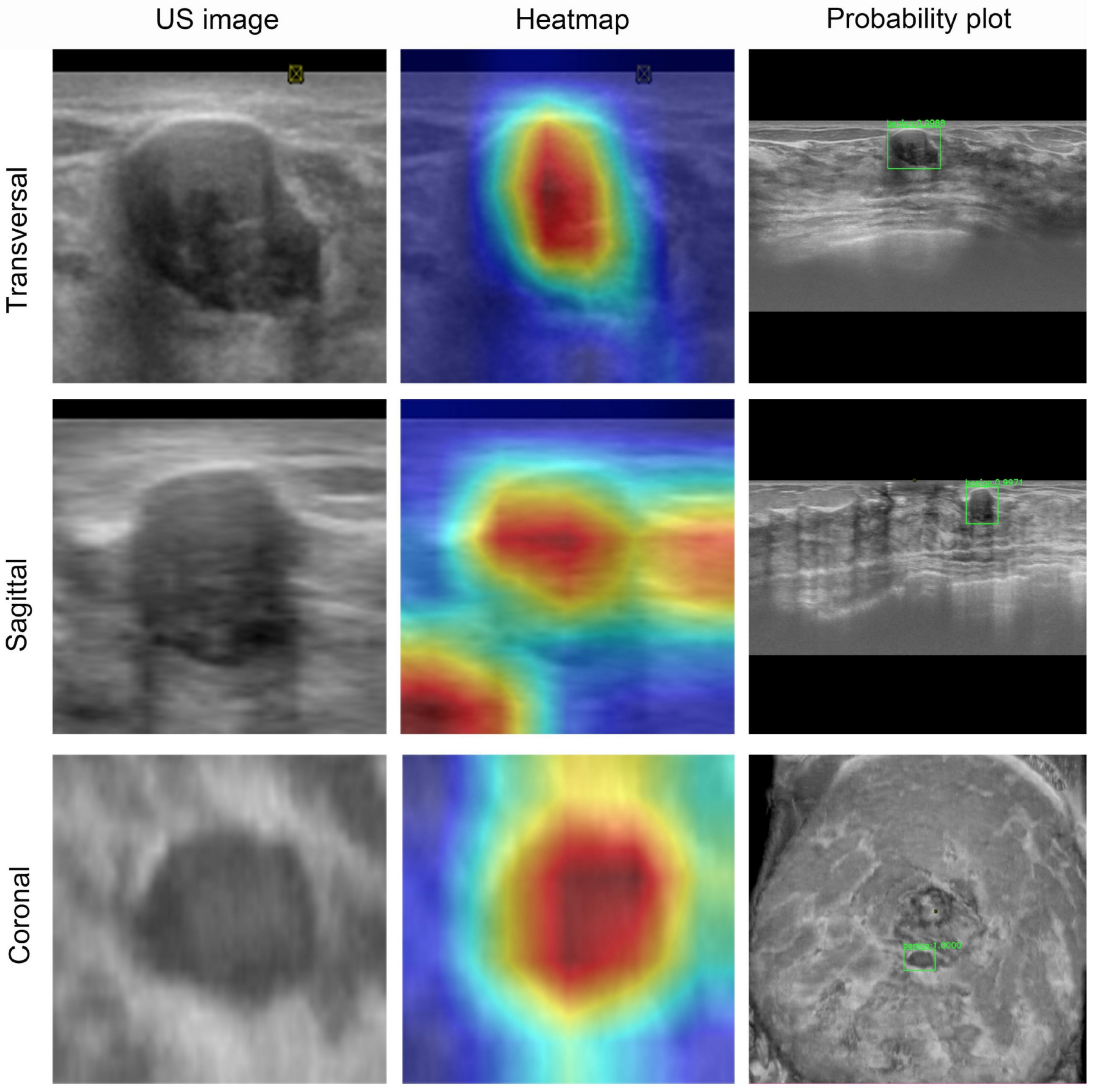
ABVS image and feature map visualization of breast tumor segmentation. ABVS image and corresponding feature map from a 24-year-old female patient presenting with a right breast mass, pathologically confirmed as fibroadenoma. The DL model predicted a benign classification (denoted by the green frame) for binary categorization, with a mean benign probability of 0.99. The superimposed heatmaps delineate diagnostically significant regions within each image. Areas depicted in warm colors (e.g., red, yellow) correspond to stronger correlations with the prediction outcome. Conversely, regions in cool colors (e.g., green, blue) indicate weaker predictive correlations. For benign tumors, the model derived its diagnostic prediction through comprehensive pixel-wise analysis within the segmented tumor region.

**Figure 5:**
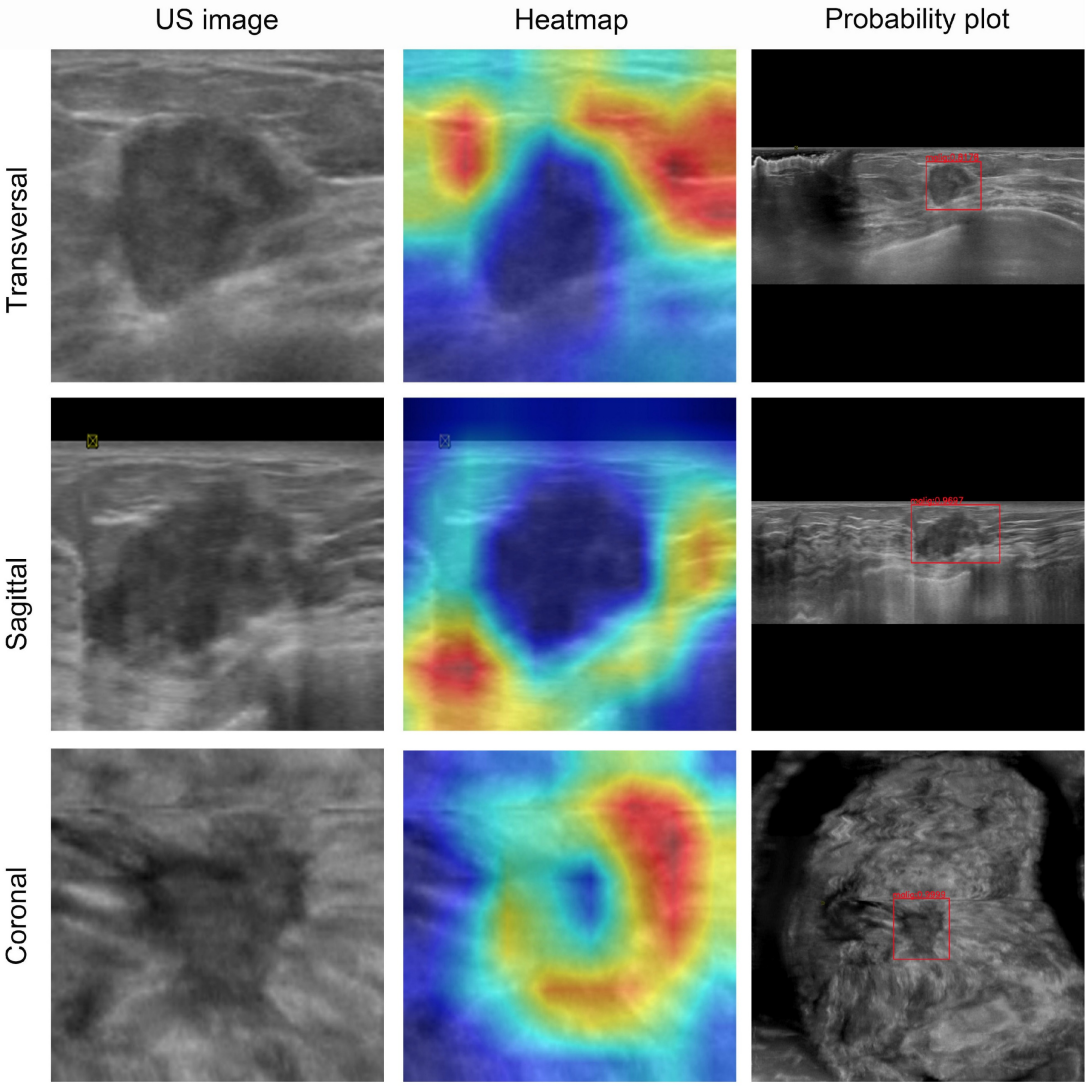
Visualization of ABVS images and feature maps for breast tumor segmentation. A 50-year-old woman presented with a palpable breast mass, which was histopathologically confirmed as invasive ductal carcinoma. The DL model predicted malignancy (indicated by a red frame for binary classification) with a mean malignancy risk probability of 0.93. These heatmaps depict the approximate locations of the lesion in each image. For malignant tumors, the model focuses more on the tumor periphery rather than the entire tumor area.

To visualize the capabilities of the DL model, the gradient-weighted class activation mapping (Grad-CAM) method was used to generate heatmaps (Figures 4 and 5). These heatmaps can highlight the most indicative areas of ABVS images, thereby interpreting the predictive mechanism of the DL model. This process reveals the contribution of each pixel in these images to the prediction of breast lesions. We observed that the DL model focused on the region where the lesion intersected with the surrounding breast glands. The basis of this prediction can assist radiologists in understanding the rationale behind the decisions made by the DL model.

### Comparison of diagnostic performance between the DL model and radiologists

Radiologists with varying levels of experience observed greater accuracy in assessing breast US lesions on three-plane US images compared to two-plane US images (Table 4). The accuracy of diagnosis by radiologists 5 and 6 was higher than that of radiologists 1 - 4. In the independent evaluation of lesions without the assistance of DL, senior radiologists demonstrated greater sensitivity and specificity than junior radiologists (94.4% vs. 60.2%; 96.8% vs. 78.2%; P < 0.05; respectively). Moreover, the sensitivity of US diagnosis by senior radiologists (94.4%) was superior to that of middle radiologists (89.8%). Compared with each individual radiologist, the ResNet50 model achieved systematically better sensitivity and specificity than junior radiologists in the internal test set (P < 0.05) and reached the level of senior radiologists with high reliability and accuracy (Table 5).

**Figure 6:**
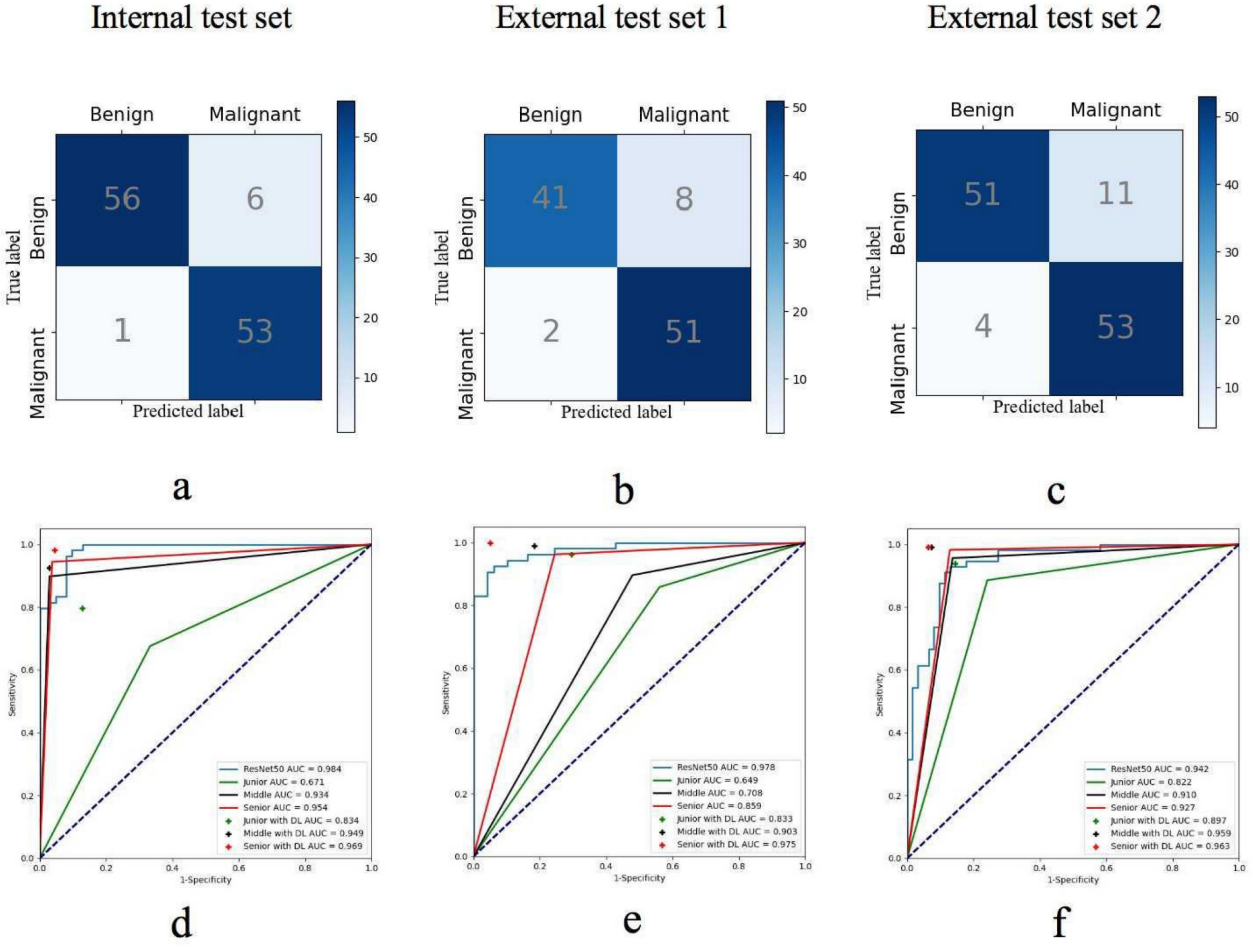
The confusion matrices for the DL model predicting breast cancer in **(a)** the internal test set, **(b)** external test set 1, and **(c)** external test set 2. The receiver operating characteristic (ROC) curves illustrate the performance of the DL model and radiologists groups groups across varying experience levels with versus without DL assistance in **(d)** the internal test set, **(e)** external test set 1, and **(f)** external test set 2.

Upon incorporating the DL method for a second diagnosis in the internal test set, there was a notable improvement in the diagnostic sensitivity of junior radiologists, which increased significantly from 60.2% to 79.7%. Similarly, the specificity improved from 78.2% to 88.7% (both P < 0.05). In contrast, the sensitivity and specificity of the middle and senior radiologists remained comparable to those in the first diagnosis, with no statistically significant differences observed (all P > 0.05). Furthermore, the diagnostic accuracy of all six radiologists improved significantly, enhancing the diagnostic performance of junior radiologists in terms of accuracy (from 69.9% to 84.4%, P < 0.05) (Figure 6).

Among all the radiologists, the reading time of the senior radiologists was shorter than that of the junior radiologists. The reading time of all the radiologists in the DL-assisted mode was shorter than that in the non-DL mode (P < 0.05). For all the radiologists, the average reading times with and without the DL-assisted mode were 14.4 seconds and 36.5 seconds, respectively. Table 5 provides a comprehensive overview of the specific changes in each diagnostic index for the six radiologists when the DL model was used. The results clearly indicate that the integration of the DL model positively enhances the diagnostic capabilities of radiologists.

### Interobserver agreement in the test set

We compared the agreement between the DL model and the six radiologists in the internal test set. For the binary classification of benign and malignant lesions, the DL model demonstrated almost perfect agreement (κ=0.809 and 0.810, 95% CI: 0.701 - 0.916 and 0.702 - 0.918) with the senior radiologists (5 and 6), substantial agreement (κ=0.755 and 0.792, 95% CI: 0.635 - 0.875 and 0.680 - 0.904) with the middle radiologists (3 and 4), and moderate to mild agreement (κ=0.222 and 0.454, 95% CI: 0.055 - 0.389 and 0.295 - 0.613) with the junior radiologists (1 and 2), respectively. The senior radiologists exhibited substantial agreement with the middle radiologists. The details are presented in Table 6.

## Discussion

The lack of consensus among inter- and intra-readers in ABVS examinations is widely recognized, and significant overlap exists in the US imaging features of benign and malignant lesions 8. The efficacy of radiological decision-making relies on both the expertise and experience of the radiologist, as well as their workload 35,36. We developed and validated a DL model for predicting breast cancer risk by analyzing and learning US features derived from ABVS images. Utilizing multiple image planes, this DL model closely replicates the standard clinical breast US scanning protocol and diagnostic reasoning. A comparative performance analysis demonstrated that the DL model achieved significantly superior diagnostic accuracy compared to junior radiologists. Furthermore, the diagnostic accuracy of the six radiologists improved significantly when utilizing the DL model as an assistive tool. Patryk et al. 36 employed a deep CNN with ABVS for breast lesion detection and classification, reporting a sensitivity of 90.9% and an accuracy of 91.0%, achieving near-perfect agreement with ground truth and performing comparably to human readers. Our study demonstrated diagnostic accuracy comparable to those reported by Wang et al. 37. The application of this novel DLN based on ABVS images holds potential for enhancing the diagnostic performance of junior radiologists. Collectively, these results indicate that the DL model can effectively evaluate breast lesions with diagnostic efficacy comparable to that of experienced radiologists.

The application of ABVS is associated with prolonged reading times and an increased rate of false-negative lesion identification. Research by Yang et al. 26 demonstrated that a significantly shorter reading time can be achieved without compromising diagnostic performance for both novice and experienced readers utilizing the concurrent-reading protocol. Our study yielded comparable findings. The proposed approach effectively facilitates the identification of suspicious lesions within ABVS datasets and provides valuable insights for accurate lesion classification, thereby contributing to significant improvements in diagnostic outcomes. The reading time for all participating radiologists was reduced in the DL-assisted mode relative to the non-DL mode, underscoring the utility of DL assistance for both junior and senior radiologists. The integration of DL models into clinical practice may serve as a dependable adjunct for experienced radiologists, offering supplementary insights, reducing diagnostic time expenditure, and furnishing expert-level guidance to junior radiologists.

The primary objective of DL application in this domain is to optimize clinical workflows and enhance diagnostic accuracy. Evaluating US images traditionally involves a time-consuming and iterative process. DL algorithms, in contrast, efficiently process extensive volumes of image data without fatigue-related degradation, demonstrating high throughput and stability throughout the diagnostic procedure. Furthermore, DL excels in recognizing complex patterns, rendering it particularly suitable for image interpretation tasks demanding the capture of nuanced details, analogous to human neural network capabilities 38. Specifically, the DL model demonstrates robust capabilities in the automated identification and delineation of breast lesions 39. Beyond diagnostic assistance, the model precisely localizes lesions and characterizes their extent, thereby augmenting the interpretability of clinical findings. Integration of DL models into US workstations offers potential for real-time radiological assistance. Deployment of DL technology as an auxiliary tool can improve diagnostic accuracy, particularly benefiting less experienced radiologists. Moreover, DL model implementation addresses challenges stemming from resource disparities. In developed regions characterized by high clinical workloads, DL models can mitigate escalating medical demands. Similarly, in resource-limited remote settings, DL models help mitigate geographic disparities in the distribution of specialized medical expertise and personnel.

In clinical practice, radiologists can continually and dynamically observe lesion evolution and three-dimensional characteristics while integrating multiple clinical parameter 40. To simulate the radiologist's clinical workflow, the DL model was developed using ABVS images, enabling multi-planar lesion visualization during routine examinations. The developed DL model demonstrated comparability and generalizability during validation, along with superior diagnostic accuracy. An additional advantage of this DL model is its inherent robustness. The model was constructed utilizing a diverse dataset comprising 1,373 cases collected from three distinct hospitals. These images were acquired by various operators employing different imaging devices. This heterogeneity in geographic origin, case characteristics, and imaging sources enhances the model's reproducibility. The AUC for the internal test set of the DL model was 0.984, and for the external test set 1, it was 0.978; for external test set 2, it was 0.942. These results indicate that the model exhibited outstanding performance in accurately identifying breast cancer risk.

There are several limitations in this study. First, the DL model was developed exclusively using grayscale US images; incorporating multimodal images data, such as color Doppler and elastography, may enhance the model performance. Second, the distribution of lesions across diagnostic classifications was uneven. Future studies should aim to include a broader spectrum of breast lesions to ensure more comprehensive representation of diverse pathological tumor types and to enrich the database. Third, subsequent research should investigate the potential of DL models for classifying molecular subtypes of breast cancer and for accurately characterizing BRCA1/2 mutation status. Further exploration is also needed regarding the optimal integration of DL models into routine clinical workflows. Advancing these research directions holds significant potential for enhancing the diagnostic capabilities of DL models in breast cancer and facilitating personalized treatment strategies.

## Conclusions

The DL model utilizing ABVS images demonstrates expert-level capability in discriminating between benign and malignant breast lesions. Radiologist performance, particularly among junior radiologists, is significantly enhanced when assisted by the DL model, as evidenced by improved diagnostic accuracy and reduced interpretation time. This DL approach fundamentally transforms conventional breast ultrasound practices by facilitating efficient, automated screening and classification of breast tumors.

## Figures and Tables

**Table 1:** Clinical and imaging characteristics of the training set, validation set, and test set.

	Training set (n = 832)		Validation set (n = 204)		Internal test set (n = 116)		External test set 1 (n = 102)		External test set 2 (n = 119)	
	M	B	M	B	M	B	M	B	M	B
Number of lesions (n)	386	446	95	109	54	62	53	49	57	62
Age (years) (mean ± SD, range)	58.1 ± 12.8 (20 - 95)	43.6 ± 12.9 (18 - 79)	57.8 ± 11.7 (29 - 84)	44.9 ± 13.5 (20 -75)	59.1 ± 12.3 (30-79)	41.5 ± 12.0 (18-67)	54.5 ± 12.0 (37-89)	43.3 ± 12.9 (18-77)	56.5 ± 11.4 (40-85)	42.5 ± 12.5 (18-61)
Age										
< 40 years (n)	37	187	6	37	4	30	25	13	14	22
≥ 40 years (n)	349	259	89	72	50	32	28	36	43	40
Lesion size (mm) (mean ± SD, range)	33.5 ± 19.3 (7 - 130)	18.8 ± 9.9 (4 - 91)	29.6 ± 14.1 (10 - 76)	19.4 ± 12.2 (4 - 83)	29.2 ± 11.4 (13 - 62)	18.5 ± 8.1 (8 - 49)	21.8 ± 6.77 (10-40)	20.5 ± 10.5 (6-60)	22.3 ± 13.4 (12-73)	19.7 ± 11.4 (7-58)
Lesion size										
T1 (< 20 mm)	81	296	20	68	13	42	14	28	16	35
T2 (20 - 50 mm)	226	142	64	36	35	19	37	20	37	24
T3 (> 50 mm)	79	8	11	5	6	1	2	1	4	3
Histology types										
Fibroadenoma		255		68		44		25		56
Adenosis		122		29		14		6		5
Other benign lesions**^a^**		69		12		4		18		1
Invasive ductal carcioma	248		48		42		41		48	
Ductal carcinoma in situ	53		19		1		2		4	
Other malignant lesions**^b^**	85		28		11		10		5	

^a^ Includes hyperplasia, benign phyllodes tumours, papillomas, inflammation, and cysts. **^b^** Includes mucinous carcinoma, invasive lobular carcinoma, malignant phyllodes tumor, and invasive carcinoma of no specific type. M = Malignant; B = Benign. External test set 1: B Hospital. External test set 2: C Hospital

**Table 2:** Performance of different models based on the three-plane data feature fusion of ABVS images.

Model	Sensitivity	Specificity	PPV	NPV	Accuracy	AUC (95% CI)	F1 score
VGG19	0.870	0.919	0.904	0.891	0.897	0.964 (0.933-0.985)	0.887
DenseNet161	0.926	0.887	0.877	0.932	0.905	0.972 (0.950-0.988)	0.901
ResNet101	0.870	0.903	0.887	0.889	0.888	0.956 (0.929-0.988)	0.879
ResNet50	0.982	0.903	0.898	0.983	0.940	0.984 (0.965-0.995)	0.938

**Table 3:** Performance of the ResNet50 model based on single, double and the three planes of ABVS images in test set.

Plane	Sensitivity	Specificity	PPV	NPV	Accuracy	AUC (95% CI)	F1 score
Transverse	0.870	0.855	0.839	0.883	0.862	0.946 (0.916-0.977)	0.855
Sagittal	0.796	0.919	0.896	0.838	0.862	0.952 (0.919-0.984)	0.843
Coronal	0.796	0.952	0.935	0.843	0.879	0.938 (0.895-0.975)	0.860
Feature fusion 1	0.889	0.903	0.889	0.903	0.897	0.963 (0.938-0.989)	0.889
Feature fusion 2	0.982	0.903	0.898	0.983	0.940	0.984 (0.965-0.995)	0.938
Feature fusion 2**^a^**	0.962	0.837	0.864	0.954	0.902	0.978 (0.951-0.994)	0.911
Feature fusion 2**^b^**	0.930	0.823	0.828	0.927	0.874	0.942 (0.902-0.978)	0.876

Feature fusion 1: transverse and sagittal feature fusion; Feature fusion 2: transverse, sagittal and coronal feature fusion **^a^** external test set 1**^b^** external test set 2

**Table 4:** Comparison of diagnostic performance among the six radiologists at different levels, according to internal test set base on the transverse and sagittal two-plane and transverse, sagittal and coronal three-plane.

		Accuracy (%)	Sensitivity (%)	Specificity (%)	PPV (%)	NPV (%)	AUC (95% CI)
Two-plane	R1	60.3 (70/116)	75.9 (41/54)	46.8 (29/62)	55.4 (41/74)	69.0 (29/42)	0.614 (0.511-0.716)
	R2	73.3 (85/116)	59.3 (32/54)	85.5 (53/62)	78.0 (32/41)	70.1 (53/75)	0.724 (0.628-0.819)
	R3	87.9 (102/116)	74.1 (40/54)	100.0 (62/62)	100.0 (40/40)	81.6 (62/76)	0.870 (0.797-0.943)
	R4	95.7 (111/116)	98.1 (53/54)	93.5 (58/62)	93.0 (53/57)	98.3 (58/59)	0.958 (0.917-1.000)
	R5	91.4 (106/116)	81.5 (44/54)	100.0 (62/62)	100.0 (44/44)	86.1 (62/72)	0.907 (0.844-0.971)
	R6	94.8 (110/116)	98.1 (53/54)	91.9 (57/62)	91.4 (53/58)	98.3 (57/58)	0.950 (0.905-0.995)
Three-plane	R1	65.6 (76/116)	66.7 (36/54)	64.5 (40/62)	62.1 (36/58)	69.0 (40/58)	0.656 (0.555-0.756)
	R2	74.1 (86/116)	53.7 (29/54)	91.9 (57/62)	85.3 (29/34)	69.5 (57/82)	0.728 (0.633-0.824)
	R3	91.4 (106/116)	83.3 (45/54)	98.4 (61/62)	97.8 (45/46)	87.1 (61/70)	0.909 (0.846-0.971)
	R4	96.6 (112/116)	96.3 (52/54)	96.8 (60/62)	96.3 (52/54)	96.8 (60/62)	0.965 (0.927-1.000)
	R5	95.7 (111/116)	92.6 (50/54)	98.4 (61/62)	98.0 (50/51)	93.8 (61/65)	0.955 (0.910-1.000)
	R6	95.7 (111/116)	96.3 (52/54)	95.2 (59/62)	94.5 (52/55)	93.8 (59/61)	0.957 (0.915-1.000)

Data represent the percentages, data in parentheses are used to calculate percentages. BI-RADS, Breast Imaging Reporting and Data System; R, radiologist; PPV, positive predictive value; NPV, negative predictive value; AUC, area under the receiver operating characteristic curve; CI, confidence interval.

**Table 5:** Comparison of diagnostic performance and reading time between the groups of radiologists at different levels with and without DL-assisted.

Different levels of radiologist group		Index	Without DL	With DL	P1	P2
Junior	R1	Reading Time (s)	41.4 ± 9.3	17.0 ± 5.7		<0.001
		Sensitivity	66.7	77.8	0.026	1.000
		Specificity	64.5	79.0	<0.001	<0.001
	R2	Reading Time (s)	39.7 ± 8.6	18.2 ± 8.4		0.048
		Sensitivity	53.7	81.5	<0.001	0.003
		Specificity	91.9	98.4	0.228	0.027
Middle	R3	Reading Time (s)	36.1 ± 5.4	14.4 ± 4.6		<0.001
		Sensitivity	83.3	87.0	0.182	0.617
		Specificity	98.4	98.4	0.371	/
	R4	Reading Time (s)	38.2 ± 6.5	15.6 ± 3.8		<0.001
		Sensitivity	96.3	98.1	0.680	1.000
		Specificity	96.8	96.8	0.680	/
Senior	R5	Reading Time (s)	33.0 ± 6.8	10.8 ± 5.2		<0.001
		Sensitivity	92.6	96.3	0.680	0.617
		Specificity	98.4	93.5	0.371	0.249
	R6	Reading Time (s)	30.9 ± 2.0	10.3 ± 1.6		<0.001
		Sensitivity	96.3	100	0.671	0.479
		Specificity	95.2	98.4	1.000	0.479

P1 values indicate a comparison between the AI model and the different levels of radiologist groups without AI assistance. P2 values indicate a comparison between the the different levels of radiologist group with AI assistance and without AI assistance. DL = Deep Learning

**Table 6:** Interobserver agreement among six radiologists and ResNet50 model.

	Kappa value (95% CI)					
	R1	R2	R3	R4	R5	R6
ResNet50	0.222 (0.055 - 0.389)	0.454 (0.295 - 0.613)	0.755 (0.635 - 0.875)	0.792 (0.680 - 0.904)	0.809 (0.701 - 0.916)	0.810 (0.702 - 0.918)
R1		0.314 (0.242 - 0.386)	0.315 (0.239 - 0.391)	0.222 (0.137 - 0.307)	0.249 (0.167 - 0.331)	0.303 (0.140 - 0.466)
R2			0.504 (0.422 - 0.586)	0.419 (0.336 - 0.502)	0.428 (0.344 - 0.512)	0.510 (0.432 - 0.588)
R3				0.755 (0.694 - 0.816)	0.805 (0.749 - 0.861)	0.733 (0.675 - 0.791)
R4					0.844 (0.794 - 0.894)	0.844 (0.794 - 0.894)
R5						0.861 (0.814 - 0.908)

## Abbreviations

ABVS: Automated breast volume scanner
AUC: Area under the receiver operating characteristic curve
BI-RADS: Breast Imaging Reporting and Data System
CNN: Convolutional neural network
DL: Deep learning
DLNs: Deep learning networks
HHUS: Handheld ultrasound
NPV: Negative predictive value
PPV: Positive predictive value
SD: Standard deviation
3D: Three-dimensional
